# Gene modelling and annotation for the Hawaiian bobtail squid, *Euprymna scolopes*

**DOI:** 10.1038/s41597-023-02903-8

**Published:** 2024-01-06

**Authors:** Thea F. Rogers, Gözde Yalçın, John Briseno, Nidhi Vijayan, Spencer V. Nyholm, Oleg Simakov

**Affiliations:** 1 Department of Neuroscience and Developmental Biology, Division of Molecular Evolution and Development, University of Vienna, Vienna, Austria; 2 Department of Molecular and Cell Biology, University of Connecticut, Storrs, Connecticut USA

**Keywords:** Genome evolution, Sequence annotation

## Abstract

Coleoid cephalopods possess numerous complex, species-specific morphological and behavioural adaptations, e.g., a uniquely structured nervous system that is the largest among the invertebrates. The Hawaiian bobtail squid (*Euprymna scolopes*) is one of the most established cephalopod species. With its recent publication of the chromosomal-scale genome assembly and regulatory genomic data, it also emerges as a key model for cephalopod gene regulation and evolution. However, the latest genome assembly has been lacking a native gene model set. Our manuscript describes the generation of new long-read transcriptomic data and, made using this combined with a plethora of publicly available transcriptomic and protein sequence data, a new reference annotation for *E. scolopes*.

## Background & Summary

Coleoid cephalopods (octopus, squid, cuttlefish) comprise a molluscan clade characterised by an abundance of complex morphological and behavioural adaptations. For instance, they possess a uniquely structured nervous system that is the largest among invertebrates, enabling exceptional camouflaging ability^1–4^. Many cephalopod clades also evolved a multitude of novel organs such as the light organ in the bobtail squids^5–7^. The genetic basis behind these innovations remains understudied due to the lack of high-quality genomes and gene annotations. So far, only a few chromosomal-scale genomes of cephalopods have been published^8–11^ and, due to their large size (about 3 Gb in octopus and over 5 Gbp in many squid or cuttlefish species^10,12^), the gene annotation has been lagging behind.

The Hawaiian bobtail squid *Euprymna scolopes* has been at the centre of cephalopod molecular research, primarily as a model for symbiotic association studies for over 30 years^13,14^. This symbiosis entails an association of the bioluminescent bacterium *Vibrio fischeri* with the light organ of the squid host. Origin of the light organ is estimated to be relatively recent (within the past 80 million years^15^) and specific to this lineage of the bobtail squids.

More recently, *E. scolopes* has also become a central model for genome evolution research^8–10,16^ These studies have identified genome-wide rearrangement events^10^ and putatively novel regulatory landscape associated with them^8^. These recent genomic insights pave the way for further understanding of coleoid cephalopod gene regulation and genomic evolutionary trends that have been hypothesised to be associated with some key coleoid innovations.

Moreover, *E. scolopes* pioneered bobtail squids in general as emerging fruitful model systems for molecular biology thanks to their small body size, relatively easy maintenance protocols^17,18^ and emerging transgenic approaches^19^.

As such, the recently published chromosomal-scale genome of *E. scolopes*^8^ was a big step forward to making this model more broadly accessible. However, the main persisting bottleneck in this resource has been the lack of proper gene models. Gene annotation was initially published in the original publication of scaffold-level *E. scolopes* genome^12^ and this annotation has been transferred to the HiC-scaffolded genome in the most recent publication^8^, however, no new gene annotation was performed on this assembly.

This manuscript describes an ongoing effort to alleviate this bottleneck by creating and refining gene annotation in the *E. scolopes* genome using a plethora of publicly available transcriptomic and protein sequence data^12,16^ with newly generated long-read transcriptomic sequencing (Table 1). PacBio Iso-Seq sequencing yielded 195,212 reads, with at least 98% of reads mapped to the genome per sample. BRAKER2 predicted 39,008 gene models and 40,590 transcripts in total, which is considerably more than the previous annotation with 24,378 models (Table 2). Further comparative analyses between closely related bobtail squid genomes^20,21^ will help validate them.

**Table 1:** Samples used for BRAKER2 gene annotation and modelling.

Sample	Data type
Testes	PacBio Iso-Seq (long read) RNA-seq
Hectocotylus (A1)	PacBio Iso-Seq (long read) RNA-seq
Skin	PacBio Iso-Seq (long read) RNA-seq
Left optic lobe	PacBio Iso-Seq (long read) RNA-seq
Central brain	PacBio Iso-Seq (long read) RNA-seq
Left white body	PacBio Iso-Seq (long read) RNA-seq
Left gill	Illumina (short read) RNA-seq
Right gill	Illumina (short read) RNA-seq
Hectocotylus (A1)	Illumina (short read) RNA-seq
B1 arm (first arm left of hectocotylus)	Illumina (short read) RNA-seq
B4 arm	Illumina (short read) RNA-seq
Right tentacle	Illumina (short read) RNA-seq
Skin	Illumina (short read) RNA-seq
Left optic lobe	Illumina (short read) RNA-seq
Suboesophageal lobe	Illumina (short read) RNA-seq
Central brain	Illumina (short read) RNA-seq
Left white body	Illumina (short read) RNA-seq
Mantle	Illumina (short read) RNA-seq
Central core	Illumina (short read) RNA-seq
Testes	Illumina (short read) RNA-seq
Ovaries	Illumina (short read) RNA-seq
*Doryteuthis pealeii*	Protein hints file
*Octopus bimaculoides*	Protein hints file
*Nautilus pompilius*	Protein hints file
*Pecten maximus*	Protein hints file
*Branchiostoma floridae*	Protein hints file

Each row represents a single sample or species protein file. All tissue samples are from *E. scolopes*. Note all PacBio Iso-Seq and Illumina RNA-seq samples used were from male individuals except the ovary sample. Illumina short read RNA-seq samples were published (and mapped as in^16^) and protein hints files were publicly available from^10,40–42^.

**Table 2:** Number of gene models and orthogroups shared between other mollusc species in the new and previous *E. scolopes* gene annotation.

Annotation	Total number of gene models	Number of orthologs *Doryteuthis pealeii*	Number of orthologs *Octopus bimaculoides*	Number of orthologs *Pecten maximus*
Rogers *et al*. (2023)	39,008	11,733	11,098	8,858
Belcaid *et al*. (2019)	24,378	11,526	10,696	9,366

**Fig. 1:**
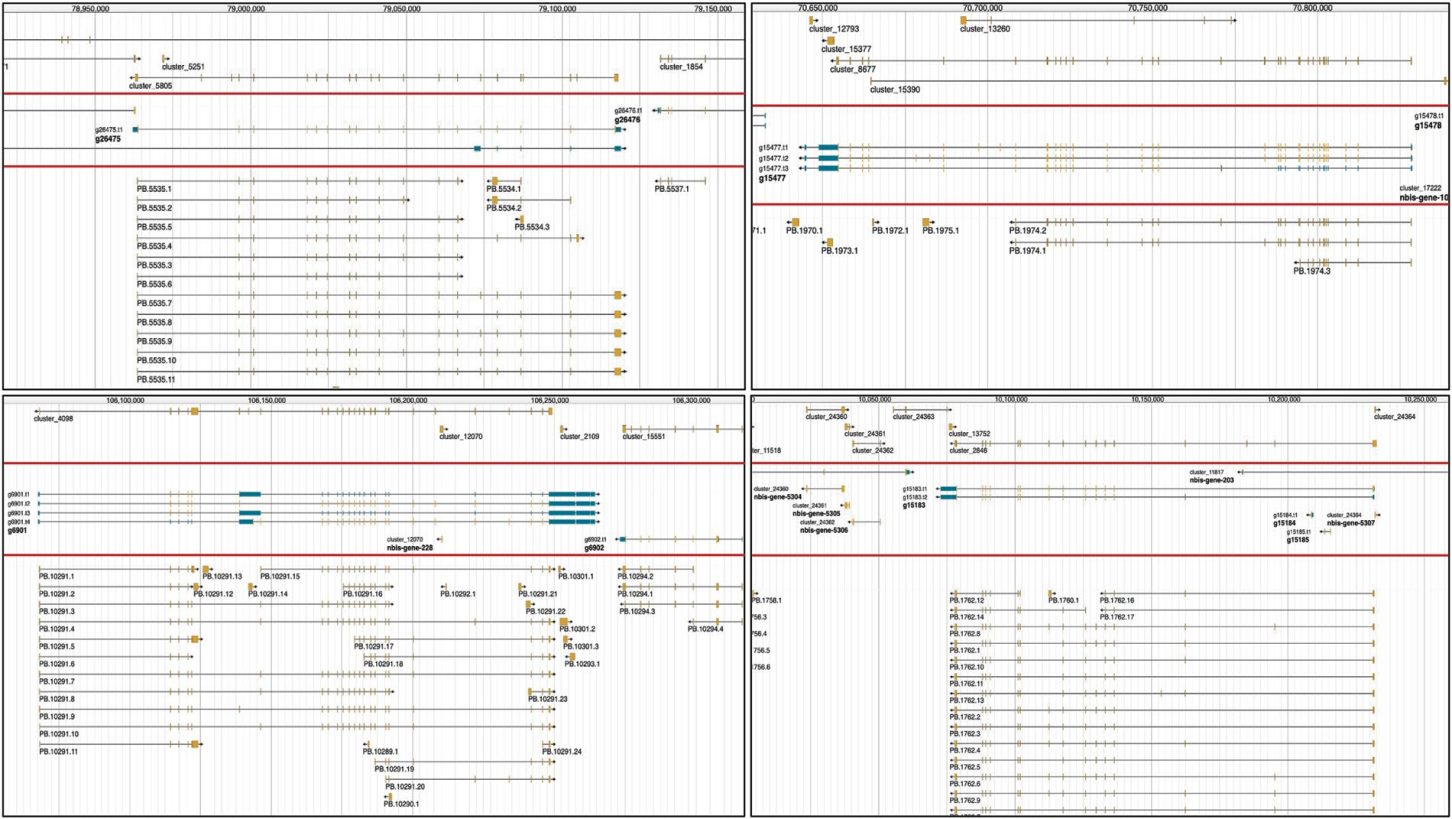
**Screenshots of the previous and new gene annotations and PacBio Iso-Seq data from the** ***Euprymna scolopes*** **genome browser**. Red lines separate tracks on the genome browser: Top; Belcaid *et al*. (2019) gene annotation, middle; Rogers *et al*. (2023) gene models^55^, bottom; new PacBio Iso-Seq data. Scale bars in bp are at the top of each screenshot. Yellow indicates exons and blue in the Rogers *et al*. (2023) annotation represents UTRs. The new gene models shown here have the following annotations according to NCBI BLASTP^56^ g6901; sodium bicarbonate transporter-like protein 11 isoform X2, g15477; phosphorylase b kinase regulatory subunit alpha (skeletal muscle isoform), g26475; E3 ubiquitin-protein ligase MGRN1, g15183; CDK5 and ABL1 enzyme substrate. The *Euprymna *genome browser can be found at: http://metazoa.csb.univie.ac.at:8000/euprymna/jbrowse.

The new annotation provided many improvements of individual loci. Examples of improvements to the gene annotation as seen on the *E. scolopes* genome browser are presented in Fig. 1. The main advantage of the latest annotation is also the addition of UTRs to the gene models. In total, 18,296 and 18,890 genes and 19,611 and 20,276 transcripts have 5’ UTR and 3’ UTR tags assigned to them, respectively. The average length of the 5’ UTRs and 3’ UTRs was 1842 and 1785 bp respectively. While this is likely to be an underestimate of the real UTR length, this annotation provides for an important improvement to help increase the quantification of scRNA-seq in cephalopod^22–24^ as well as regulatory genomics studies^8^, through proper identification of transcription start sites.

## Methods

### Biological materials

All adult animal experiments were conducted in compliance with protocol number A18–029 approved by the Institutional Animal Care and Use Committee, University of Connecticut. Adult *E. scolopes* were collected from Maunalua Bay, Oahu, Hawaii (21°16’51.42”N, 157°43’33.07”W), and were transported to the University of Connecticut where they were maintained in recirculating artificial seawater. Animals were euthanized and tissues were sampled for RNA as described below.

### RNA extraction and sequencing

Animals were anaesthetised using 2% ethanol, organs were dissected and submerged in TRIzol™. Samples were then flash frozen in liquid nitrogen and stored at −80 °C. RNA was extracted within a week of flash freezing. RNA was extracted from the hectocotylus (A1), testes, skin, left optic lobe, and central brain from one male individual of *E. scolopes*. Additionally, RNA was extracted from the left white body of a different *E. scolopes* male. Samples were processed using the TRIzol™ manufacturer’s protocol, and homogenised in 1 ml TRIzol™ in a freestanding 2 mL bead-beating tube with 0.1 mm Zirconia/Silica beads using a Qiagen PowerLyzer. The final RNA pellet was washed three times with 75% ethanol at 4 °C and resuspended in 30 µL of nuclease-free water. Next, the samples were treated with Ambion’s Turbo DNA-free kit, and their quality was assessed using an Agilent 5300 Fragment Analyzer system. RIN scores and electropherograms for extracted RNA used for PacBio Iso-Seq can be seen in supplementary Figure S1 and Table S1. Libraries were prepared using an oligo dT primer to transcribe only the polyA-mRNA and then sequenced using the PacBio Iso-Seq Sequel II 30hrs mode on one SMRTcell at the Vienna Biocenter Core Facility.

### Processing and mapping of PacBio Iso-Seq data

PacBio reads were filtered for bq (barcode call quality) less than 45, reads were demultiplexed and primers were removed using Lima v.2.7.1^25^. The PacBio Iso-Seq data was then processed according to the bulk Iso-Seq workflow found at: https://isoseq.how/getting-started.html. Here, PolyA tails and concatemers were identified and removed and hierarchical clustering was performed using IsoSeq. 3 v.3.8.2^25^ with standard parameters. Next, reads were aligned to the *E. scolopes* reference genome (BioProject number PRJNA661684^8^) using the pbmm2 align command with default parameters in pb-assembly v.0.0.8^25^. Bam files for each sample were then merged in Samtools v.1.7^26^ to use for gene modelling. The merged files were then collapsed in IsoSeq. 3 in order to view them on the *E. scolopes* genome browser (http://metazoa.csb.univie.ac.at:8000/euprymna/jbrowse).

### Gene modelling and annotation

Gene modelling was performed using BRAKER2^27–39^ on the softmasked *E. scolopes* reference genome^8^. The PacBio Iso-Seq data for *E. scolopes* newly generated here, published Illumina RNA-seq data (mapped as in^16^) for *E. scolopes*, and publicly available protein hints files from *Doryteuthis pealeii*^10^, *Octopus bimaculoides*^10^, *Nautilus pompilius*^40^, *Pecten maximus*^41^ and *Branchiostoma floridae*^42^ were used for training (Table 1). Both Illumina RNA-seq and PacBio Iso-Seq were inputted into BRAKER2 using the —bam option, whilst protein files were specified with the–prot_seq option. Note all Illumina RNA-seq and samples inputted into BRAKER2 were male except for one female gonad sample. Once BRAKER2 had finished, untranslated regions (UTRs) were then added by running BRAKER2 again with and the Iso-Seq and RNA-seq, as well as the –addUTR = on and –skipAllTraining parameters, pointing to the augustus.hints.gtf file in the first BRAKER2 run using –AUGUSTUS_hints_preds^27–29,35,36,43–45^. The output of the second BRAKER2 run, gushr.gtf, was formatted for downstream analyses using the TSEBRA scripts fix_gtf_ids.py and rename_gtf.py^46^ and a custom perl script. We then sought to complement this with the previously available mapping of transcripts^12^. For this, we used GMAP version 2023–07–20 to map available Belcaid *et al*.^12^ CDS sequences to the genome. Next, bedtools v2.30.0^47^ was used to intersect CDS regions of gushr.gtf models with the mapped Belcaid *et al*.^12^ CDS regions. We then selected Belcaid *et al*.^12^ models with two or more coding exons and that had at least 75% of their coding exons not matching BRAKER2 models and added these to the gushr.gtf annotation using a custom perl script. Lastly, CDS and exon lines were added to the GTF using another perl script.

### Generation of coding sequence, protein sequence and protein annotation files

Protein sequence and coding sequence files were generated by running gffread from GffRead v0.12.7^48^ on the reformatted gushr.gtf annotation file. Interproscan v5.62–94.0^49^ with default parameters was used to perform annotation of the protein sequence file.

### Quality checking of gene models

The previous^12^ and new gene annotations for *E. scolopes* were assessed for completeness using BUSCO v.5.4.5^50^ with metazoa_odb10 in protein mode and OMArk v.0.3.0^51^ with the ancestral clade Lophotrochozoa. OrthoFinder v.2.5.5^52^ was used to count the number of orthogroups shared between each annotation and *Doryteuthis pealeii*^10^, *Octopus bimaculoides*^10^ and *Pecten maximus*^41^. The number of single- and multi-exon genes with and without protein annotation was calculated using a custom perl script along with the interproscan.tsv output file from Interproscan.

## Data Records

The raw, demultiplexed PacBio Iso-Seq data underlying these analyses have been deposited in the NCBI database under Bioproject PRJNA99482^53,54^. The gene annotation, coding sequence, protein sequence and protein annotation files can be found on GitHub under: https://github.com/TheaFrances/E.scolopes-V2.2-BRAKER2-gene-annotation^54^ and Dryad under: 10.5061/dryad.nk98sf7xz^55^.

## Technical Validation

The crucial improvement over the previous annotation^12^ was the addition of de-novo gene models on the latest chromosomal-scale assembly^10^ including UTR prediction and detection of many isoforms. In terms of protein coding content, our current annotation is, as expected, not substantially exceeding the BUSCO scores of the previous one^12^ (Tables 2 and 3). However, the OMArk results show improvement in all categories (Table 4). Additionally, the new annotation presents less missing BUSCOs compared to the previous gene annotation, highlighting the benefit of *de novo* gene modelling on the latest chromosomal-scale assembly. We further note that manual inspection of missing BUSCOs has yielded many loci that are present in single copies in *E. scolopes* genome and represented in the gene model set, but are highly divergent at the sequence level. Such genes may encode for proteins with accelerated evolutionary rates in coleoid cephalopod genomes. Further construction of an accurate coleoid cephalopod-focused single copy orthology dataset will thus be needed to properly assess genome completeness in these genomes. Note that the increase in the number of duplicated BUSCO and OMArk scores is a result of the addition of transcripts (isoforms) per gene present in the new annotation.

**Table 3:** BUSCO scores for the new and previous *E. scolopes* gene annotation (lineage Metazoa).

Annotation	Complete BUSCO	Single BUSCO	Duplicated BUSCO	Fragmented BUSCO	Missing BUSCO
Rogers *et al*. (2023)	83.2% (794)	76.1% (726)	7.1% (68)	10.8% (103)	6.0% (57)
Belcaid *et al*. (2019)	86.1% (822)	83.3% (795)	2.8% (27)	6.8% (65)	7.1% (67)

**Table 4:** OMArk scores for the new and previous *E. scolopes* gene annotation (ancestral clade used: Lophotrochozoa).

Annotation	Complete OMArk	Single OMArk	Duplicated OMArk	Missing OMArk
Rogers *et al*. (2023)	95.6% (2268)	70.5% (1673)	25.1% (595)	4.4% (105)
Belcaid *et al*. (2019)	93.34% (2215)	77.12% (1830)	16.22% (385)	6.66% (158)

The number of orthogroups shared between the new annotation and *D. pealeii*, and shared between the new annotation and *O. bimaculoides*, increased compared to the orthogroups shared with the old annotation and these species. There were fewer orthogroups shared between *P. maximus* and the updated annotation compared with *P. maximus* and Belcaid *et al*.^12^ (Table 2). We find that 30,766 models were multi-exon genes, and 9,824 models were single-exon. While it is possible that single-exon models were false-positive predictions, we still were able to annotate 4,811 of them with Interproscan (compared to 25,413 in the multi-exon gene set), and thus decided to retain them in our prediction set.

The current chromosomal-scale reference genome contains many gaps (over 30%, genome assembly statistics reported in Supplementary Table 1 from Schmidbaur *et al*.^8^). BUSCO scores for the genome assembly, using metazoa_odb10 are as follows: complete 83.4%, (single: 82.9%, duplicated: 0.5%), fragmented: 10.2%, missing: 6.4%. Parallel efforts are yielding an almost gap-free reference assembly, on which the gene models presented in this paper will be transferred and improved further, potentially including the missing exons and decreasing the “missing” BUSCO count even more.

### Supplementary information

Supplementary Information

## Data Availability

List of commands run and scripts used are available on GitHub under: https://github.com/TheaFrances/E.scolopes-V2.2-BRAKER2-gene-annotation^54^.
