# Supplementary material for: Gene modelling and annotation for the Hawaiian bobtail squid, Euprymna scolopes
Source: Sci Data. 2024 Jan 6;11:40. doi: 10.1038/s41597-023-02903-8 (PMC10771462; doi:10.1038/s41597-023-02903-8)
Supplement: Supplementary file 1 — Supplementary Information [file 41597_2023_2903_MOESM1_ESM.docx]

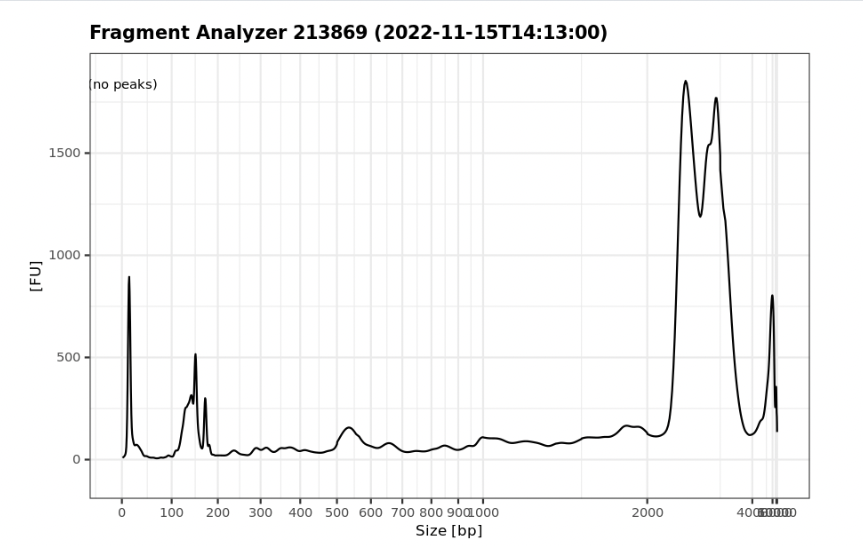


**Left white body**


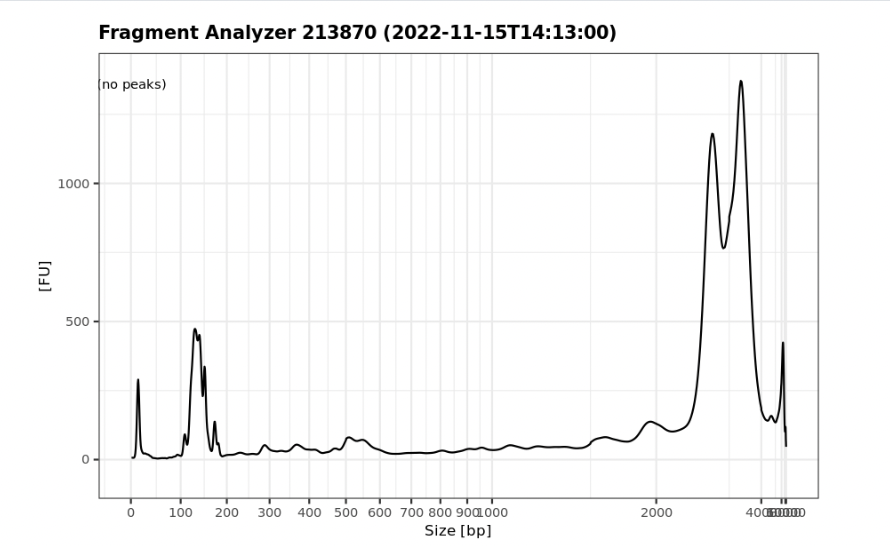

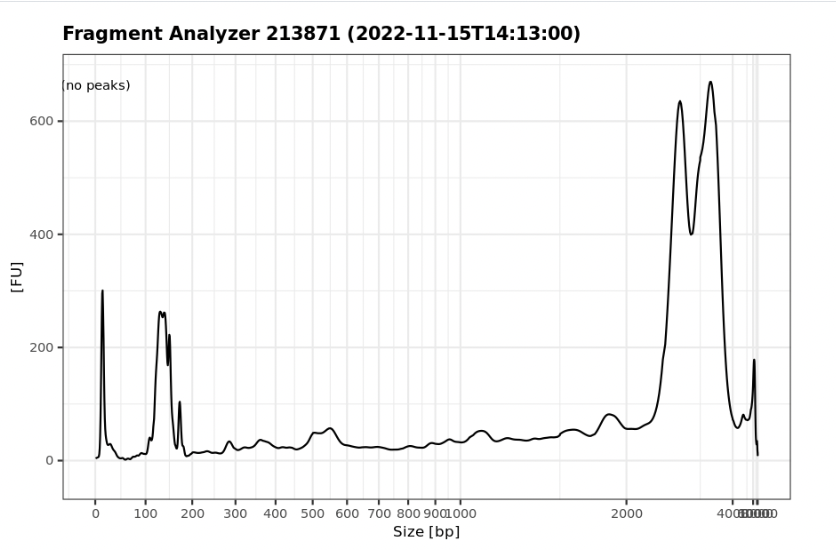


**Hectocotylus (A1)**

**Central brain**

D.

E.

F.


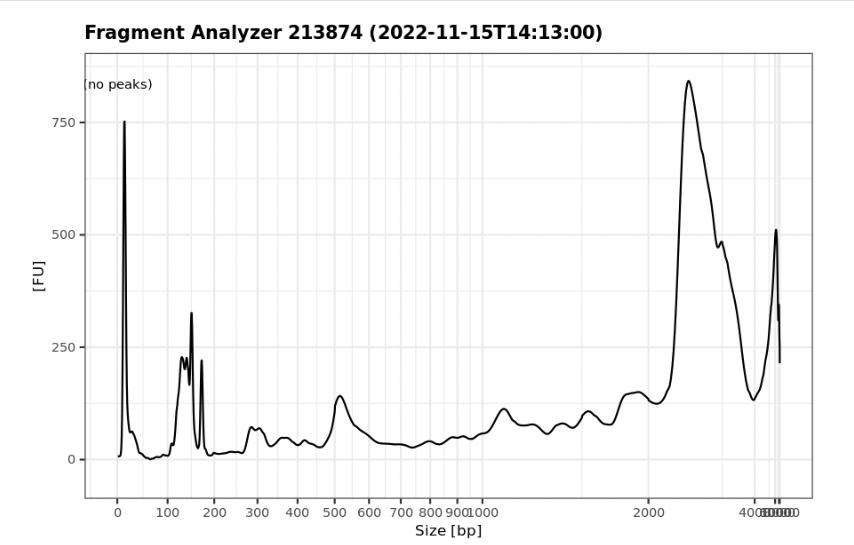

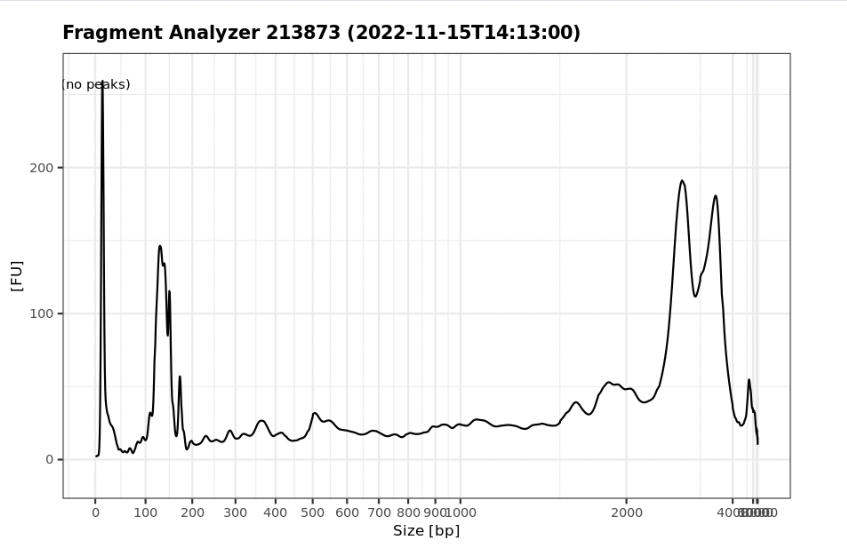

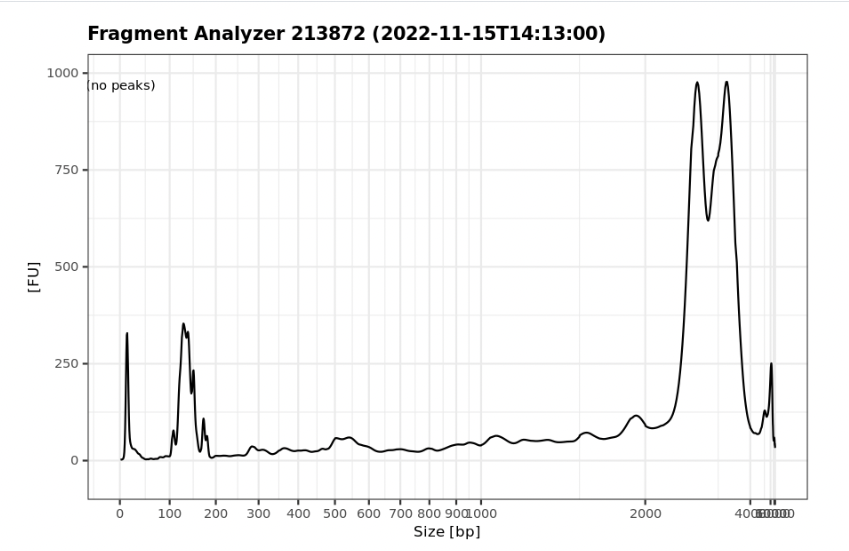


**Left optic lobe**

**Skin**

**Testes**

**Figure S1. Fragment analyzer electropherograms for extracted RNA used for PacBio Iso-Seq.**

| **Sample** | **RIN score** |
| --- | --- |
| Left white body | 8.9 |
| Central brain | 8.6 |
| Hectocotylus (A1) | 8 |
| Left optic lobe | 8.3 |
| Skin | 6.4 |
| Testes | 8.4 |

**Table S1. RIN scores for extracted RNA used for PacBio Iso-Seq.**
